# Role of a Heat Shock Transcription Factor and the Major Heat Shock Protein Hsp70 in Memory Formation and Neuroprotection

**DOI:** 10.3390/cells10071638

**Published:** 2021-06-29

**Authors:** Olga G. Zatsepina, Michael B. Evgen’ev, David G. Garbuz

**Affiliations:** Laboratory of Molecular Mechanisms of Biological Adaptation, Engelhardt Institute of Molecular Biology of the Russian Academy of Sciences, 119991 Moscow, Russia; olzacepina@yandex.ru (O.G.Z.); misha672011@yahoo.com (M.B.E.)

**Keywords:** molecular chaperones, Hsp70, heat shock factor 1 (HSF1), stress, memory formation, long-term potentiation, ischemic injury, neurodegenerative disorders

## Abstract

Heat shock proteins (Hsps) represent the most evolutionarily ancient, conserved, and universal system for protecting cells and the whole body from various types of stress. Among Hsps, the group of proteins with a molecular weight of 70 kDa (Hsp70) plays a particularly important role. These proteins are molecular chaperones that restore the native conformation of partially denatured proteins after exposure to proteotoxic forms of stress and are critical for the folding and intracellular trafficking of de novo synthesized proteins under normal conditions. Hsp70s are expressed at high levels in the central nervous system (CNS) of various animals and protect neurons from various types of stress, including heat shock, hypoxia, and toxins. Numerous molecular and behavioral studies have indicated that Hsp70s expressed in the CNS are important for memory formation. These proteins contribute to the folding and transport of synaptic proteins, modulate signaling cascades associated with synaptic activation, and participate in mechanisms of neurotransmitter release. In addition, HSF1, a transcription factor that is activated under stress conditions and mediates Hsps transcription, is also involved in the transcription of genes encoding many synaptic proteins, whose levels are increased in neurons under stress and during memory formation. Thus, stress activates the molecular mechanisms of memory formation, thereby allowing animals to better remember and later avoid potentially dangerous stimuli. Finally, Hsp70 has significant protective potential in neurodegenerative diseases. Increasing the level of endogenous Hsp70 synthesis or injecting exogenous Hsp70 reduces neurodegeneration, stimulates neurogenesis, and restores memory in animal models of ischemia and Alzheimer’s disease. These findings allow us to consider recombinant Hsp70 and/or Hsp70 pharmacological inducers as potential drugs for use in the treatment of ischemic injury and neurodegenerative disorders.

## 1. Introduction

The ability of organisms to survive in constantly changing environmental conditions, to reproduce, and to occupy new ecological niches largely depends on the functioning of the nervous system, which is critical for the input of new information, its processing, and motor response necessary for rapid avoidance of danger. Rapid behavioral responses may help to reduce or completely prevent environmental stressors, such as temperature increases, dehydration, changes in the chemical composition of aquatic organisms, and predator attacks [1]. Successful reactions to avoid dangerous influences are based on memory [2,3,4]. The ability to remember and form conditioned reflexes to avoid danger has been shown for a wide variety of organisms, such as nematodes, molluscs, crustaceans, *Drosophila*, and mammals [4,5,6,7,8,9,10]. Notably, the formation of memory occurs in parallel with the activation of the metabolic systems in the response to stress, i.e., an adaptive response that protects individual cells and the whole organism from various stress factors. The adaptive response to stress also protects the synaptic network (the memory substrate) from the damaging effects of different stress factors and during neurodegeneration processes in aging or proteinopathies [11].

The most universal system of protection from any stressful influence is represented by a group of genes encoding so-called “heat shock proteins” (Hsps) or stress proteins. These proteins provide a certain degree of cell resistance to hyperthermia, hypoxia, oxidative stress, toxins of various types, radiation, etc. [12]. A group of proteins with a molecular weight of 70 kDa (HSP70) plays a special role in ensuring the survival of the cell in stressful conditions and maintaining normal metabolism. Hsp70 plays an important role in protecting nerve cells during ischemia and neurodegenerative diseases. The experimental data available at the moment allow us to postulate the crucial role of Hsp70 in memory formation, nervous system development, and maintaining the functioning of the nervous system during aging.

## 2. Materials and Methods

A comprehensive literature review was conducted to identify and critically evaluate studies analyzing the possible relationships between Hsp70 and memory or neuroprotection. The PubMed, PubMed Central, and Scopus databases were searched for related research articles. Selection and data collection of study were carried our form blind and independently by all three coauthors. Keywords for searching included the following: Hsp70, heat shock factor 1 (HSF1), stress, memory, short-term potentiation, long-term potentiation, short-term memory, long-term memory, ischemic injury, neuroprotection, neurodegenerative disorders, and development of nervous system. Discrimination by year of publication was not used. Only articles already published or accepted for publication were used for citation in the review.

In addition to the standard search in the PubMed and Scopus databases, article search was based on work with Entrez-direct utility (ftp://ftp.ncbi.nih.gov/entrez/entrezdirect/ accessed on 12–13 April 2021), which provides an API-like access to NCBI PubMed database. Entrez-direct is able to fetch all articles whose titles matching a regular expression. The data obtained is in MEDLINE format, containing all the basic metadata of article containing abstract text and year of publication. Subsequent analysis of article metadata included keyword extraction with Rapid Automatic Keyword extraction algorithm (RAKE), implemented in Udpipe R package (https://ufal.mff.cuni.cz/udpipe accessed on 15 April 2021), calculating keyword co-occurrence rates, and time-dependent trend analysis of normalized keywords and MEDLINE terms frequencies. The data acquired allowed us to work with most relevant scientific articles in this paper.

## 3. Mechanisms of Hsp70 Regulation and Memory Formation

**Figure 1:**
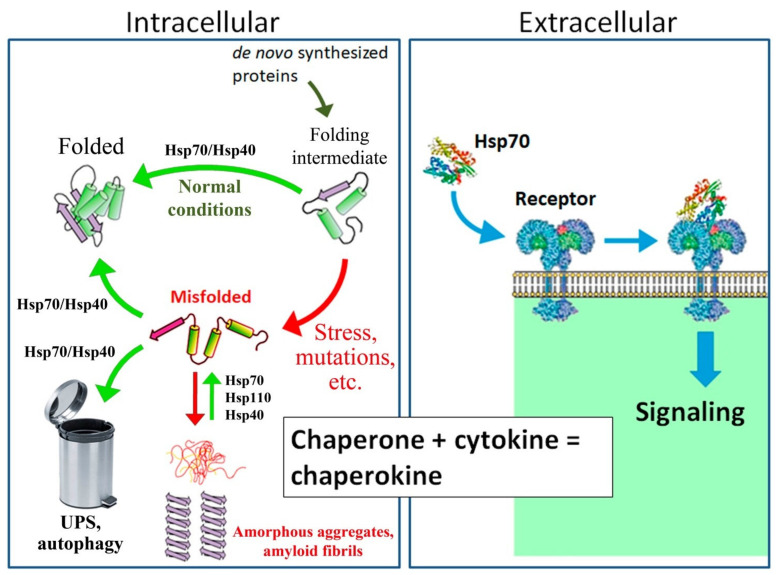
Mechanism of action of intracellular and secreted proteins of the Hsp70 family. As an intracellular housekeeping protein, Hsp70, in cooperation with Hsp40 and other co-chaperones, folds and sorts newly synthesized proteins in cells under normal conditions. Under proteotoxic stress and in the case of certain mutations, misfolded proteins accumulate in the cytosol. Hsp70, in cooperation with co-chaperone Hsp40, restores the native conformation of partially denatured proteins and directs irreversibly damaged proteins to the ubiquitin–proteasome system (UPS) or lysosomes by autophagy. In addition, Hsp70 in combination with Hsp110 and Hsp40 promotes the dissolution of protein aggregates, such as α-synuclein [12,17,18,19,20]. As a secreted protein, Hsp70 is recognized by TLR2/4 and CD91 receptors and participates in the regulation of innate immunity, similar to classical cytokines; hence, Hsp70 is often called a “chaperokin” [21,22,23,24,25,26,27,28].

The mechanism of action of Hsps is based on their ability to interact with the hydrophobic regions of partially denatured proteins, thereby preventing their aggregation and promoting the recovery of their native conformation. Hsp70 also mediates the correct folding and transport of newly synthesized proteins under normal conditions (Figure 1) [11]. Proteins with similar activities are called molecular chaperones. The most versatile molecular chaperones are proteins belonging to the Hsp70 family (each member with a molecular weight of approximately 70 kDa). Hsp70 is one of the most conserved cellular proteins and is found in the cells of all studied organisms at all stages of phylogeny [13,14]. The Hsp70 family, as well as other families of molecular chaperones, includes a group of proteins whose synthesis is induced by stress (inducible Hsp70) and several proteins that are synthesized mainly under normal physiological conditions (constitutive Hsc70) [15]. Hsc70 primarily facilitates the folding and intracellular transport of proteins, while Hsp70 is primarily involved in refolding or degradation of proteins that have been partially damaged or denatured under stress conditions (Figure 1). Notably, Hsp70 is a strong inhibitor of apoptosis [16].

The induction of heat shock protein synthesis is triggered under stress that causes abnormal protein conformation [29]. The transcriptional induction of Hsp genes is mediated by a family of transcription factors called “HSFs” (heat shock factors) [30,31]. When the concentrations of partially denatured proteins, cAMP, and calcium ions increase in the cytosol (due to heat shock or other types of stress), the transcription factor HSF1 is trimerized and phosphorylated. Activated HSF1 binds to heat shock elements (HSEs) located in Hsp promoters, resulting in a ten- to hundred-fold increase in transcription intensity [30,31,32,33]. In mammals, constitutive Hscs are expressed with the participation of another transcription factor, HSF2, which is active under normal conditions [34].

Some Hsps, in particular Hsp70, in addition to serving as molecular chaperones in intracellular processes, are secreted into the intercellular space, where they play a role in intercellular communication. Secreted Hsps are recognized by a number of pattern-recognizing receptors (TLR2, TLR4, and others) (Figure 1). Therefore, extracellular Hsp70 was initially characterized as one of the damage-associated molecular patterns (DAMPs), intracellular molecules that are released from damaged or dying cells due to trauma or infection and activate the innate immune response via pattern-recognizing receptors located mostly on neutrophils and macrophages [35]. Subsequently, it has been suggested that the pro-inflammatory effects of exogenous Hsp70 are explained by its contamination with lipopolysaccharide (LPS), which is capable of inducing the reaction of macrophages and neutrophils, even when LPS is present in trace amounts [36]. However, the results obtained using Hsp70 isolated from eukaryotic expression systems (for example, the baculovirus system) and free from contamination by LPS and bacterial proteins indicated that exogenous Hsp70 may have an anti-inflammatory effect. This effect involves suppression of the secretion of pro-inflammatory cytokines, reactive oxygen species and NO by neutrophils and macrophages and reduction in the nuclear transport of NF-kB [21,22,23,24,25,26,27].

The formation of memory at the molecular level occurs in several stages. First, short-term memory (STP) is formed, which does not require the synthesis of new proteins. It is believed that the formation of short-term memory involves the activation of adenylate cyclase and the production of cAMP, which leads to the activation of protein kinase A (PKA). Activated PKA phosphorylates a wide range of proteins, including potassium and calcium channel subunits, leading to the strengthening of pre-existing synaptic connections [37].

Long-term memory requires, in addition to posttranslational modifications and activation of existing synapses, the synthesis of new proteins and the formation of new synapses [37,38]. The formation of long-term memory and its consolidation requires the repetition of learning stimuli, which leads to a prolonged increase in the level of cAMP and phosphorylation of the transcription factor cAMP response element (CRE)-binding protein (CREB), as well as the induction of several genes with CRE motifs (CREB targets) in the promoter region [39,40]. The CREB-mediated response to extracellular stimuli is modulated by a set of protein kinases (PKA, CaMKII, CaMKIV, RSK2, ERK1/2, and PKC) and phosphatases (PP1 and calcineurin) [37,41]. At the next stage, both the de novo transcribed mRNAs and the resting mRNAs stored locally at the synapses are translated, further stabilizing the synapse.

**Figure 2:**
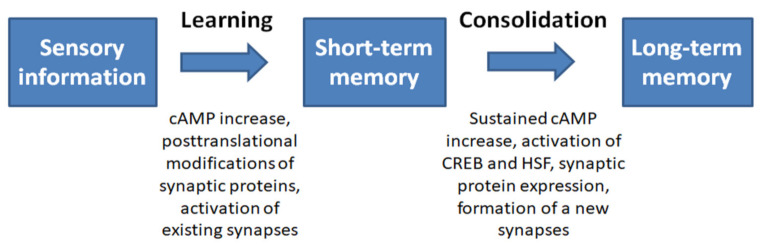
Summary of the sequence of events during memory formation [37,38,41,42].

Strengthening of both pre-existing and newly formed synaptic connections induced by brief high-frequency stimulation is referred to as long-term potentiation (LTP) [42]. LTP formation is associated with enhanced neurotransmitter release and is one of the main mechanisms underlying learning and memory [42,43]. In most synapses that support LTP, there is a postsynaptic increase in the concentration of calcium, mediated by the activation of NMDA (N-methyl-D-aspartate) receptors. An increase in calcium levels leads to the activation of CaMKII, which is expressed both in presynaptic and postsynaptic terminals. On the presynaptic side, the protein substrates for CaMKII phosphorylation include synapsin, synaptotagmin, and synaptophysin, which play key roles in the release of neurotransmitters. On the postsynaptic side, CaMKII substrates include several other proteins, such as α-actinin, PSD95, the synaptic adhesion protein densin-180, microtubule-associated protein 2 (MAP2) and neurofilament *L*. Phosphorylation of these proteins leads to cytoskeletal rearrangement and structural changes in synapses, further strengthening the connection [43]. The memorization sequence is given in Figure 2.

Stimuli that require memorization are often associated with various types of stress. Animals need to remember potentially dangerous environmental impacts (temperature changes, predator attacks, etc.) that require an adequate response (avoidance or defense), as well as needing to be aware of their own actions, remembering those that may lead to negative consequences. Most likely, through these connections during the course of evolution, the relationship between the stress response systems, in particular the mechanisms of Hsp70 induction, and memory was formed and consolidated. It has been shown that the stressful effects of moderate-intensity stressors contribute to the formation of LTP and memory [6]. It has also been demonstrated that Hsp70 family members, which are universal molecular chaperones, are involved in the processes of protein synthesis and trafficking that are necessary for the maintenance of existing synapses and the formation of new synapses. Finally, Hsp70 may act as a neuroprotector, to some extent, reducing the impact of damaging factors and ageing of the nervous system, including memory deterioration. Therefore, it is evident that the relationship between Hsp70 functions and memory formation is of fundamental interest and carries promise for the treatment of age-related neurodegenerative diseases, such as Alzheimer’s disease.

## 4. Relationship between the Stress Response System and Memory

Environmental and physiological stresses activate the transcription of *hsps* in all studied organisms mainly through activation of HSF family members [44]. In addition to HSEs (heat shock elements), which are targets of HSF1, the promoter region of mammalian *hsp70* genes contains sterol response element (SRE) motifs that mediate the induction of Hsp70 by growth factors (e.g., nerve growth factor, NGF) and a CRE motif, which is necessary for the binding of the phosphorylated form of CREB [45,46]. The presence of CRE motifs in the promoter region of *hsp70* genes suggests its possible role in the processes of neuroregulation and memory. Phosphorylated CREB is known to initiate the transcription of several genes associated with memory consolidation processes [37,47,48]. Both CREB and HSF1 are activated under stress conditions and involve the participation of several stress-induced signaling cascades (MAPK, PKA, PKC, CaMKII, and Akt) in response to an increase in Ca^2+^, cAMP, and other low-molecular-weight mediator concentrations [31,49,50,51,52,53,54]. It has been demonstrated that increased Ca^2+^ levels and activation of CaMKII and PKC are involved in both the regulation of Hsp70 transcription and memory formation [55,56].

Binding sites of the transcription factor FOXO/DAF-16 have been found in the promoter region of *Drosophila melanogaster hsp70* genes [57]. It has also been shown that FOXO/DAF-16 plays an important role in learning and memory, as well as in stress resistance in *Caenorhabditis elegans* [58,59]. Furthermore, induction of Hsp70 has been shown to be expressed when tasks related to learning and memory are undertaken (see below) [60,61,62].

To date, a large number of studies have indicated an important role of HSF1 in the processes of memory formation. For instance, it has been demonstrated that HSF1 activation leads to improved cognitive abilities [63,64] and that loss of HSF1 activity is associated with neurodegeneration [65]. Notably, the promoters of genes encoding many synaptic proteins, in particular, PSD95, synapsin I, and synaptophysin, contain canonical HSE sequences. Activation of HSF1 with 17-aminoallylgeldanamycin (17-AAG) has been shown to increase the expression of PSD95, synapsin I, synaptophysin, SAP97, and the neurotrophic factor BDNF, a key regulator of synaptic plasticity [66,67]. In addition, 17-AAG has been shown to increase the expression of Hsp70 and Hsp27 in neurons in vivo and to enhance LTP [67]. Curcumin, shown to act as an HSF1 activator, increases BDNF levels in the hippocampus and reduces memory loss in rodent models of Alzheimer’s disease (AD) [68,69]. Another study showed that activation of HSF1 and/or CREB during synaptic formation induces Hsp70 expression in postsynaptic structures [70,71,72]. Since the formation of long-term memory requires rapid synthesis of several new proteins, the participation of Hsp/Hsc70 as molecular chaperones in this process is absolutely necessary.

HSF1 also mediates the expression of β-amyloid precursor protein (APP) [73], and HSEs are found in the promoter of the APP gene. Various types of stress (HS, ethanol, treatment with sodium arsenite) lead to the activation of APP transcription. The APP protein is known to be involved in the transmission of intercellular signals and in cell adhesion, promoting contacts between neurons and the formation of new synapses [73,74].

Currently, it is known that the action of stress factors that leads to protein denaturation is not the sole pathway leading to HSF1 activation. For instance, it has been shown in *C. elegans* that serotonin, which is involved in the stress response in all animals and modulates their physiological and metabolic adaptation to adverse conditions [75,76], promotes the activation of HSF1 independent of temperature elevation [75,76,77]. Serotonin is sufficient for the short-term induction of Hsp70 and thermal tolerance. Serotonin is known to play an important role in learning and memory, and a decrease in its level in the mammalian brain leads to memory impairment [78,79].

Interestingly, Hsp70 and Hsp60 levels have been elevated in daphnia placed in water previously exposed to predators (fish) [80]. It has also been shown that Hsp70 is synthesized in the brain of a fish (*Carassius auratus*) under the influence of cortisol released at the sight of a predator. This phenomenon may be associated with the memorization of threats and the formation of a defensive response and/or avoidance reaction [81]. Similarly, repeated presentation of a food odor or a visual danger stimulus triggers Hsp70 expression in the nervous system of the crab *Chasmagnathus granulatus* [82]. In rats, the stress caused by immersion in water has been shown to cause a significant increase in the mRNA levels of Hsp70 and Hsc70 in the hippocampus [83,84]. Thus, in addition to proteotoxic forms of stress, psychophysiological stress such as a danger can cause the induction of Hsp70 family protein expression.

A role of HSP70 in memory has also been demonstrated in rodent models [56,85]. The increase in the level of Hsp70 proteins in mice and rats was detected using different training protocols. Both Hsp70 and Hsc70 are induced in the hippocampus after stress-related spatial learning (contextual fear conditioning, CFC, a behavioral paradigm based on the ability to learn and remember aversive stimuli) [60,61,62]. The concentration of Hsp70 increases in the cerebellum after a two-way avoidance task, with the maximal level expression observed during the task solution phase and a decrease after the memory consolidation phase [60,86]. However, the time of maximum Hsp70 induction after training varies depending on the study protocol. In Reference [62], a short-term increase in Hsp70 levels in the hippocampus was observed 1 h after training. The authors suggest that the rapid induction of Hsp70 in the hippocampus is due to the activation of PKA and CREB during and immediately after training [87]. Injection of recombinant Hsp70 (rHsp70) at a concentration of 0.5 mg/mL into the dorsal hippocampus immediately after exercise promoted learning and memory. At the same time, administration of rHsp70 at a concentration of 0.25 or 1 mg/mL did not affect memory consolidation. It is possible that Hsp70 at high concentrations is toxic or activates pro-inflammatory signals [88,89]. In line with this possibility, transgenic mice that constitutively express high levels of Hsp70 have previously been shown to exhibit reduced learning ability in some tests [90]. The authors suggest that a constant high level of Hsp70 causes stable changes in the structure of synapses and increase in LTP, with no further increase in LTP detected after training.

A moderate increase in temperature leading to Hsp70 induction has been shown to increase learning ability. For example, fluctuations in water temperature leading to Hsps production have a positive effect on memory formation in the pond snail *Lymnaea stagnalis* [91]. Similarly, mild HS restores memory in the *D. melanogaster* Volabo and Agnostic mutants that normally have learning and memory problems [92,93].

The relationship between stress and memory is illustrated by “Yerkes–Dodson law” [94], which postulates that, up to a certain limit, stress promotes memory, but with a further increase in the intensity of stress, the ability to remember decreases. This phenomenon may be explained by the fact that, under excessive stress, the synthesis of Hsp70 is suppressed due to the rapid activation of the stress kinases p38 and JNK, which inhibit the activity of HSF1 [95,96].

In addition to participating in protein folding, Hsp70 can modulate the activity of many signaling proteins that are involved in various regulatory cascades. Thus, Hsp70 is known to inhibit the activation of the p38/JNK stress kinases under mild stress or normal conditions [97,98]. It has also been shown that the injection of rHsp70 after CFC leads to a change in the activity of the MAPK signaling pathway, enhancing the ERK1/2 cascade while reducing the activity of p38/JNK. Furthermore, administration of rHsp70 increases the level of CREB phosphorylation in the hippocampus. Presumably, ERK1/2 activity is necessary for the phosphorylation of CREB and CRE-dependent transcription of genes critical for memory [85,99]. Additionally, ERK1/2 phosphorylate synapsin I protein, leading to the mediator release and strengthening of the existing synapses, as well as the formation of new synapses [100].

**Figure 3:**
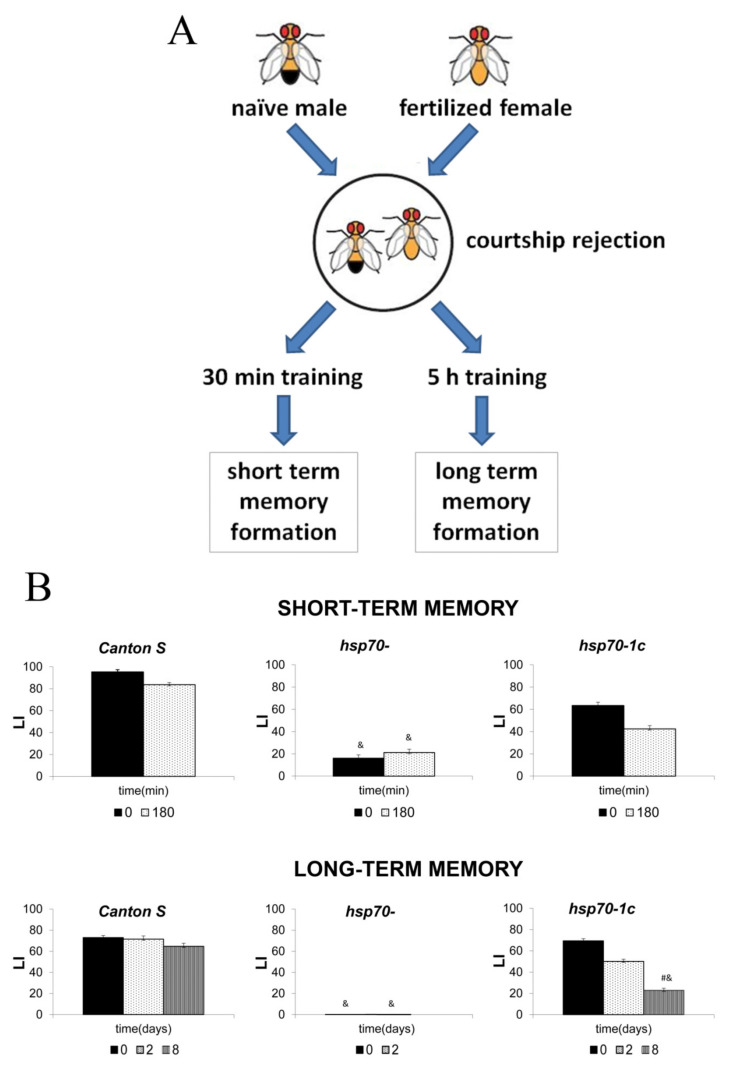
Results of an experiment on the effect of Hsp70 on memory. (**A**) A fertilized *Drosophila* female rejects the male’s advances by spraying him with an anti-aphrodisiac secretion. The male, who remembers the reaction of the fertilized female, later refuses to court fertilized females. (**B**) STM and LTM in males of the Canton S strain with six copies of the Hsp70 gene, the Hsp70-knockout strain (Hsp70-), and the strain with one restored copy of the Hsp70 gene (Hsp70-1c). The absence of Hsp70 led to a decrease in STM and loss of LTM, whereas restoring a single copy of Hsp70 restored STM and LTM, compared to the corresponding memory levels in the Hsp70-negative strain. LI refers to learning index. &, #—*p* < 0.05. [105].

Many experiments using different mouse strains with several *hsp70* family genes deleted have demonstrated their sensitivity to ischemia and shown a higher probability of neurodegenerative diseases in these mice [101,102,103,104]. However, to our knowledge, there have not been reports describing the effect of Hsp70 knockout on memory formation. These studies would significantly improve our understanding of the molecular mechanisms underlying Hsp70 involvement in memory formation and consolidation. In particular, we used the conditioned courtship suppression paradigm (CCSP) to elucidate the roles of *hsp70* genes in memory formation in a *D. melanogaster* model [105]. We found that a constitutively low level of Hsp70 is required for learning and short- and long-term memory formation in males. Strains with all six *hsp70* genes deleted demonstrated poor ability to learn and establish short-term memory (STM) and complete inability to form long-term memory (LTM) in the CCSP (Figure 3). The presence of at least one *hsp70* copy was sufficient to restore the ability to form STM and partially rescued LTM under normal conditions. A transcriptome analysis revealed that males with different *hsp70* copy numbers after courtship suppression differ significantly in the expression of several groups of genes involved in the memory process, reproduction, and immunity. Importantly, our analysis revealed a few pathways involved in memory formation and consolidation, including the cAMP signaling cascade, which depends on the presence of *hsp70* in the genome [105].

**Figure 4:**
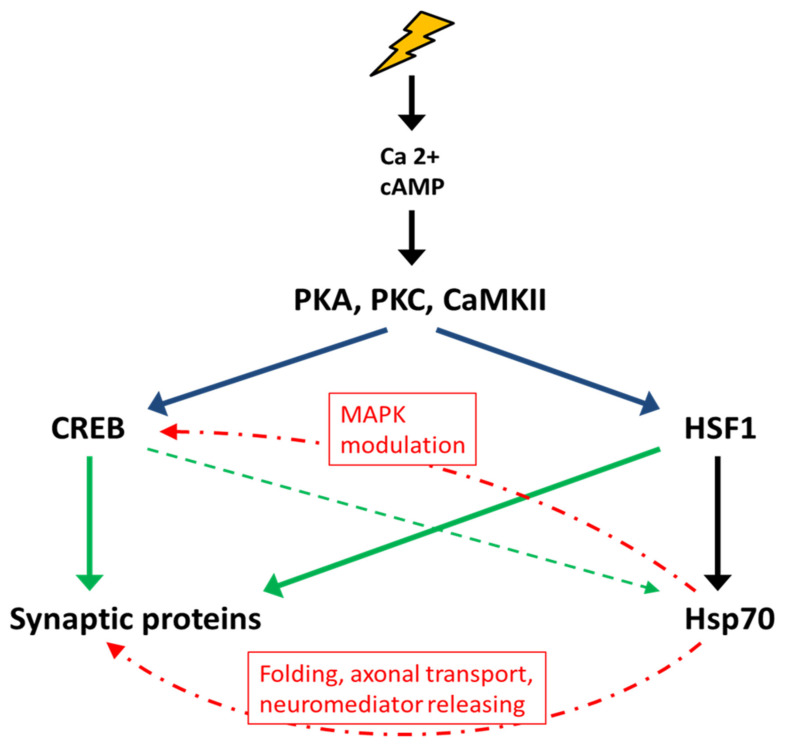
Interactions between the stress response and memory formation. Stress stimuli lead to an increase in the concentration of calcium and cAMP in the cytosol in CNS cells and the activation of protein kinases PKA, PKC, CaMKII, etc., which are involved in the regulation of the activity of transcription factors, in particular, CREB and HSF1. Both CREB and HSF1 initiate the transcription of genes encoding synaptic proteins and Hsp70. In turn, Hsp70 promotes the folding and transport of synaptic proteins and the release of neurotransmitters, activates the MAPK signaling cascade, and ensures the structural integrity of synapses by interacting with the cytoskeleton.

Generally, from the data currently available, it can be concluded that HSF1 and CREB form a joint regulatory network that determines the interaction of the stress response system and memory formation (Figure 4). Their joint participation in this interaction is necessary for the induction of proteins involved in the reorganization of existing synapses and the formation of new ones (i.e., PSD95, synapsin I, synaptophysin, SAP97, BDNF, and APP). In addition, Hsp70 family proteins are required for newly synthesized synaptic protein folding, reorganization of the synaptic actin cytoskeleton, and modulation of signaling cascades involved in memory formation.

## 5. Hsp70 is Involved in the Functioning of Synapses and Protects the Synaptic Network from Stress-Induced Damage

Hsp70/Hsc70 proteins are continuously synthesized in the nervous system under normal physiological conditions at all stages of ontogenesis (in the absence of stress) [106,107,108]. Thus, Hsc70 expression is found in preimplantation mouse embryos at the 2 blastocyst stage [109]. During subsequent development, high levels of Hsc70 are maintained in the cytosol and nuclei of neuroectoderm cells during neural plate differentiation, neural tube closure and organogenesis [110].

Several studies [106,111] have shown that the Hsc70 level is elevated in all neuronal tissues (retina, cerebellum, cortex, and brainstem) compared to that in other organs. Hsc70 levels reach 2 to 3% of the total protein content in rat spinal cord cells [112]. The constitutive synthesis of Hsp70 in the brains of mouse embryos begins much later than Hsc70 synthesis, on the 15th day of development. Importantly, in mice, Hsc70 is localized in neurons, while Hsp70 is mainly localized in glia. At the same time, glia may secrete various types of Hsps, mainly Hsp70, that are then internalized by neurons, mainly in the synaptic region. This process is activated under stress conditions, particularly by moderate temperature elevation [101,106,113,114,115]. In the postnatal period, the CNS has a high constitutive level of Hsp70 expression compared to other organs and tissues [116]. The expression of Hsp70 in the CNS correlates with a high constitutive level of HSFs [117].

A high level of Hsc70 expression has been observed in all synaptic fractions (synaptic membranes, synaptic junctions (SJs), and postsynaptic density (PSD) elements), not only in newly formed synapses but also in previously existing synapses [118]. Hsc70 is a component of synaptic vesicles and is necessary for the recirculation of synaptic vesicles in presynaptic nerve endings [119,120]. On the other hand, constitutive synthesis of Hsp70 has been observed in the SJ and PSD fractions of the forebrain. Most of the newly synthesized synaptic proteins are transported from the neuron body along the entire length of the axon. During this long-term transport along the axon, some of the neuron bodies lose their native structure. As a result of slow axonal transport, misfolded and functionally inactive proteins that enter synapses accumulate in the synaptic region. The preservation of the native structure and function of synaptic proteins is fundamentally important for the functioning of neurons [121]. Thus, high levels of Hsc70 and Hsp70 in synapses are necessary to restore and/or maintain the native conformation of the transported proteins [118]. Hsp70 also interacts with the cytoskeleton, stabilizing the structural morphology of synapses and thereby preserving synaptic transmission [122].

The expression of Hsp70 in different areas of the central nervous system increases under stress [123]. A slight rise in temperature, which induces Hsps expression, protects nerve cells from the effects of an even higher temperature, which can be lethal in nerve cells that have not been preheated. This phenomenon is called induced thermotolerance and is particularly important for poikilothermic animals [124,125]. Experiments using either nerve cell cultures transfected with Hsp70-expressing constructs or transgenic animals overexpressing Hsp70 in the CNS have shown that Hsp70 plays an important neuroprotective role [126,127,128]. Under stress conditions, synaptic activity must be maintained to prevent disruption of vital connections in the nervous system. Obviously, selective overexpression of Hsp70 in the synaptic region increases the level of synaptic protection. It has been shown that Hsp70, induced by stress, is localized to the synapses of the rat brain [118]. Another independent study has found that hyperthermia causes the translocation of Hsc70 to the synaptic region [129]. The authors suggest that Hsp/Hsc70 protects the nervous system at the functional level by supporting neurotransmission in synapses during stress. It has also been shown that chronic hypoxia induces the synthesis of Hsp70, which is necessary to maintain the expression level of presynaptic proteins. A direct interaction has been found between Hsp70 and the presynaptic protein syntaxin. Thus, heat shock-induced Hsp70 expression supports the release of neurotransmitters at elevated temperatures or during ischemia, providing synaptic homeostasis [130,131].

## 6. Hsp70 Is an Effective Neuroprotector for Brain Ischemia

Overexpression of inducible Hsp70 protects the brain against ischemia. This effect has been shown in both cell cultures and animal models of stroke [132]. The expression of transgenic Hsp70 protects primary neurons from heat shock and plays a crucial role in determining the fate of neurons after ischemia [133]. Ischemia, which leads to hypoxic nerve tissue damage, induces the induction of Hsp70 synthesis [16,134]. Ischemic stroke affects two areas: the ischemic nucleus, in which nerve cells undergo apoptosis and/or necrosis, and the “penumbra”, in which the survival of neurons is possible. In cerebral ischemia in rats, neurons expressing Hsp70 have been shown to be preferentially located in the penumbra region at the border of the viable tissue surrounding the infarct nucleus, and in the infarct region, Hsp70 has also been shown to be induced in microglia, astrocytes, and endothelial cells [16,134,135]. In transgenic mice overexpressing Hsp70, the effects of cerebral ischemia are significantly less pronounced than those in wild-type mice [136,137]. On the other hand, knocking out Hsp70 genes leads to a significant increase in the volume of affected nerve tissue in experimental focal ischemia in mice [101,102]. Geranylgeranylacetone, an inducer of Hsps synthesis, also exhibits a neuroprotective effect in animal models of ischemia and traumatic brain injury [138,139].

Endogenous Hsp70 contributes to the increased survival of neurons in ischemia due to its ability to block apoptosis and because of its anti-inflammatory properties. Hsp70 can inhibit apoptosis at both early and later stages. Thus, Hsp70 has been shown to inhibit the activation of SAPK/JNK family stress kinases, which represent the main triggers of apoptosis through the phosphorylation of p53 and the antiapoptotic protein bcl-2 [97,98]. Hsp70 also inhibits the formation of apoptosomes, involving cytochrome *c*, Apaf-1, and procaspase 9. Moreover, Hsp70 blocks apoptosis-inducing factor (AIF) translation to the nucleus [16,102,104,140]. In the case of ischemia, the trafficking of the pro-apoptotic receptor Fas and the secretion of FasL bound with Fas increase. Hsp70 inhibits Fas-mediated apoptosis by interfering with the transport of the Fas receptor from the Golgi complex to the cell surface [16]. Additionally, endogenous Hsp70 prevents the activation of the pro-inflammatory factor NF-kB and its transport to the nucleus. This effect is mediated by the interaction of Hsp70 with IkB (inhibitor of kB). As a result, overexpression of Hsp70 leads to a decrease in the production of major pro-inflammatory mediators such as NO and ROS [16,141].

The neuroprotective potential of not only endogenous Hsp70 expressed in nerve tissue cells but also exogenous (recombinant) Hsp70 in ischemia was demonstrated. When administered intranasally in mice, full-size recombinant Hsp70 effectively penetrates the brain and exhibits a pronounced anti-inflammatory effect [103,142,143]. Similarly, it has been shown that intranasal administration of Hsp70 for 5 days reduces one-half the volume of the ischemic lesion in a photothrombotic stroke mouse model [144]. This procedure reduces the apoptosis of neurons in the penumbra and significantly increases the expression of synaptophysin, which indicates the restoration of synaptic networks. In addition, exogenous Hsp70 promotes increased neurogenesis in the hippocampus after ischemic stroke [144].

A promising approach for the treatment of ischemic conditions is the creation of chimeric constructs that combine the Hsp70 gene with a protein transduction domain, such as Tat from HIV-1, which facilitates the transport of fusion protein to the central nervous system by crossing the blood-brain barrier after intravenous or intraperitoneal administration. These proteins have shown higher neuroprotective activity in mice with induced ischemia than unmodified Hsp70 [145,146,147]. At the same time, exogenous chimeric Hsp70 has been shown to stimulate neuroblast differentiation and proliferation, as well as CREB phosphorylation in the hippocampus, and to improve memory [85].

Thus, both endogenous and exogenous Hsp70 effectively protect nerve tissue, including the main “substrate” of memory, i.e., synaptic structures, from damage during ischemia. This protection allows us to recommend further research on the possibility of using both recombinant Hsp70 and inducers of endogenous Hsp70 synthesis as efficient and harmless neuroprotectors in the treatment of ischemic strokes.

## 7. Hsp70 Prevents Neurodegeneration and Promotes Memory Recovery in Alzheimer’s Disease Models

The described neuroprotective properties of Hsp70 may be of great importance for the prevention and treatment of various neurodegenerative diseases, such as Alzheimer’s disease (AD), the frequency of which has recently increased dramatically in developed countries as a result of increased life span [148,149]. The cause of neurodegeneration in many cases is proteinopathies, i.e., protein folding disorders, leading to excessive accumulation and aggregation of certain proteins in the central nervous system. These neuropathologies include Parkinson’s disease and dementia with Lewy bodies (DLB), which develop due to aggregation of α-synuclein; frontotemporal dementia (tauopathy); Huntington’s disease belonging to a group of polyglutamine disorders; and amyloidosis (Alzheimer’s disease and Creutzfeldt–Jakob disease) [150,151,152,153]. Among the listed diseases, AD is currently of the greatest socioeconomic importance. The course of sporadic and hereditary forms of AD includes prolonged deterioration of the patients’ mental condition, often lasting years. Typical symptoms are progressive memory loss and the development of dementia, and the outcome of AD is inevitably the death of patients [148,149]. More than 100 million cases of Alzheimer’s disease are predicted to be diagnosed 2050 [154]. It is assumed that the main cause for the development of AD is the accumulation in the brain of soluble and highly toxic oligomers and insoluble aggregates of the so-called Aβ-peptide, which is a product of the proteolysis of the APP protein, an important participant in intercellular communication in the nervous system [155]. Alternative hypotheses have been proposed on the basis that the key factor in the development of AD is considered to be the aggregation of hyperphosphorylated tau protein (tauopathy), chronic inflammation of brain tissues (neuroinflammation), or the accumulation of mutations in mitochondrial DNA, leading to dysfunction of the mitochondrial respiratory chain and the development of oxidative stress [156,157,158]. Currently, the main models of AD are represented by various transgenic strains of rodent species that express mutant forms of APP and β/γ secretases (proteolytic enzymes involved in the proteolysis of APP via the amyloidogenic pathway to form Aβ peptides) [159,160,161]. In these animal models, at the age of 5 to 6 months, Aβ deposition and neurodegeneration are detected in the cortex and hippocampus, with subsequent loss of cognitive function. A model of bulbectomized mice that develop all major manifestations of AD a few months after the surgical removal of the olfactory bulbs has also been considered valid [142,162,163]. The main clinical feature used in studies of mouse models of AD is spatial memory loss, usually tested through the Morris water maze, and an increase in β-amyloid levels in specific areas of the brain [164].

Several groups have shown that the chaperone properties of Hsp70 are of great promise to cure neurodegenerative diseases, including AD. The decrease in Hsp70 levels in CNS tissues observed with age may be one of the factors contributing to the accumulation of toxic Aβ oligomers and tau aggregates, increasing the risk of AD [165]. Most authors postulate the importance of a decrease in the levels of Hsp70 and Hsc70 in the development of AD [166]. Thus, according to Franklin et al. 2005, tau aggregation is largely associated with a decrease in the chaperone activity of Hsp70 and other Hsps in aged individuals. On the other hand, some authors show an increase in Hsp70 levels at the early stages of AD, with Hsp70 co-localizing with tau protein aggregates [165]. Hsp70 and Hsc70 are involved in the degradation of hyperphosphorylated tau by ubiquitinylation of the latter with the participation of the ubiquitin ligase CHIP, which increases the survival of neurons in tauopathy [165,167,168]. The phosphorylated residues on tau act as recognition sites for Hsp70, marking the protein for ubiquitinylation and subsequent proteasomal degradation by the E3 ligase CHIP [169].

There is also evidence that Hsp70 interferes with the oligomerization of purified Aβ in vitro [170]. However, Hsp70 does not reduce the toxicity of pre-oligomerized Aβ; that is it is not able to dissolve oligomers [171]. On the other hand, the introduction of an Hsp70-producing adenovirus vector into a primary culture of Aβ-expressing neurons dramatically increases the survival rate of the culture (fivefold) and reduces the level of Aβ accumulation in the cytoplasm [172].

Transgenic mice expressing a mutant APP producing Aβ peptides with a high tendency to aggregate, after crossing with transgenic mice overexpressing Hsp70 show a decrease in Aβ levels, a decrease in neurodegeneration (reduced loss of neurons and synapses), and recovery in terms of cognitive function (memory ability as measured by Morris test) compared to cross with wild-type mice [173]. This outcome is not due to a decrease in the production of Aβ but results from the activation of its phagocytosis and degradation systems via IDE (insulin-degrading enzyme, an Aβ-degrading enzyme involved in the degradation of Aβ) [173]. Induction of Hsp70 synthesis by geranylgeranylacetone in mice expressing mutant APP leads to a decrease in Aβ levels and memory recovery in the Morris test [174]. In addition to the IDE, the MAPK pathway activation also participates in the effect of Hsp70 to reduce Aß40/42 production in APP- transgenic mice [175].

The induction of Hsp70 with the subsequent development of a neuroprotective effect is mediated by the trimerization of HSF1 under the influence of stress factors or specific chemical compounds. HSF1 may also have an alternative neuroprotective effect, independent of trimerization, Hsp accumulation, and activation of signaling pathways, including the CaMKII, PKA, casein kinase II, and PI-3K-Akt pathways. Overexpression of HSF1 completely blocked mutant huntingtin-mediated toxicity in rat cerebellar granule neuronal (CGN) cultures. The authors suggest that this effect is mediated by the class III histone deacetylase (HDAC) SIRT1, which is known to have strong neuroprotective effects, through a direct interaction between monomeric HSF1 and SIRT1 in the nuclei of neurons [176].

Models of bulbectomized mice that develop all major manifestations of AD a few months after surgical removal of the olfactory bulbs are also considered to be valid [142,162,163]. Surgical removal of the olfactory bulbs (bulbectomy) in mice causes the development of several symptoms characteristic of AD, including increased levels of Aβ, neurodegeneration in the temporal cortex and hippocampus; deficient serotonin, acetylcholine, and glutamatergic system levels; and memory loss [162,163]. In addition, changes in the Aβ level and other manifestations of AD follow a certain pattern after bulbectomy. The concentration of Aβ in the brain increases significantly 1.5–2 months after bulbectomy (5- to 6-fold compared to sham-operated mice) but then gradually decreases, reaching a minimum 6 months after surgery. Then, the concentration of Aβ begins to increase further (7- to 8-fold compared to the sham-operated animals 12 months after the operation). Changes in the level of Aβ correlate with the ability of experimental animals to learn and remember in the Morris water test [177]. In bulbectomized animals, spatial memory is significantly reduced 1.5 months after surgery compared to sham-operated mice but is subsequently restored (6 months after bulbectomy, corresponding to a minimal concentration of Aβ). In addition, a strong negative correlation between the concentration of Aβ (and the degree of memory loss) and the level of endogenous Hsp70 in the brains of bulbectomized mice has been observed. In this study, the Hsp70 level begins to increase shortly after the removal of the olfactory bulb, reaches a maximum 6 months after surgery, and then declines. By the twelfth month, it becomes significantly lower than the level of Hsp70 in sham-operated mice. Furthermore, the maximum level of Hsp70 corresponds to the period of maximal memory recovery and a decrease in the Aβ concentration. These findings suggest that increased Hsp70 synthesis in the brain after bulbectomy is a compensatory mechanism that is activated in response to brain injury and to some extent contributes to the temporary recovery of cognitive functions. The mechanism of the observed protective action of endogenous Hsp70 may be realized by the endogenous Hsp70 facilitation of blocking tau and Aβ aggregation and promoting their degradation [165,172,174,178].

Hsp70 may have important implications and clinical prospects not only in the case of AD but also in other proteinopathies leading to neurodegeneration. The neuroprotective effect of Hsp70 overexpression has been shown in many models of Parkinson’s disease in vitro and in vivo. Heat shock-mediated or geldanamycin-induced induction of Hsp70 can prevent α-synuclein-induced cell death in yeast, *Drosophila* and mouse models of PD [179,180,181]. Thus, it has been shown that Hsp70 prevents the formation of toxic α-synuclein oligomers that subsequently aggregate into insoluble fibril-forming Levi bodies, which is considered the main cause of Parkinson’s disease [182,183]. In addition, Hsp70 in combination with Hsp40 and Hsp110 participates in the dissolution of α-synuclein amyloid fibrils to form α-synuclein monomers in vitro [17,18,19]. Finally, Hsp70 stimulates autophagocytosis of α-synuclein fibrils and, facilitated by Hsp40 and E3 ubiquitin ligase CHIP, degradation of alpha-synuclein monomers via ubiquitin–proteasomal pathway [20]. Additionally, Hsp70-mediated disaggregation and autophagy are necessary for the quality control of polyglutamine proteins, including huntingtin, whose aggregation is the cause of Huntington’s disease [184]. Induction of Hsps by geldanamycin derivatives in vitro inhibits aggregation of the prion protein PrPSc; thus, prion infection develops faster in *Hsp70−/−* knockdown mice than in wild-type mice after PrPSc inoculation [185]. In summary, the effects of intracellular Hsp70 on the prevention of protein aggregate formation in the development of neuropathies, tau, alpha-synuclein, and polyglutamine proteins are realized through three main pathways: folding/degradation of monomers and prevention of the formation of oligomers and dissolution of aggregates and autophagy. Currently, chemical inducers of Hsp70 synthesis, such as geldanamycin derivatives, radicicol, or geranylgeranylacetone, are considered possible therapeutic drugs for various neurodegenerative diseases [186].

**Figure 5:**
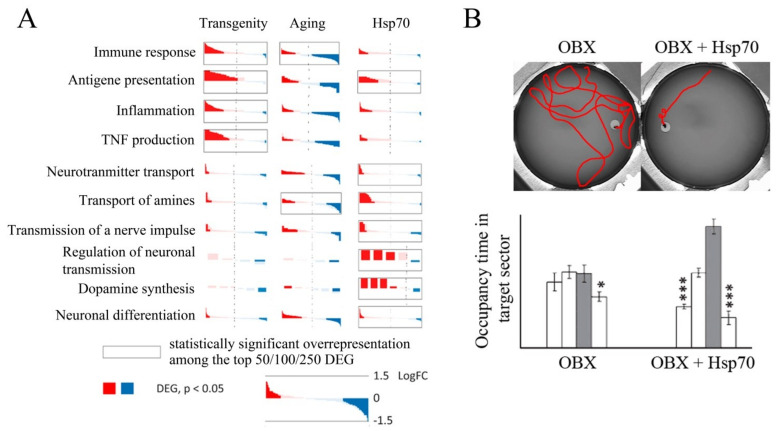
(**A**) Differentially expressed genes (DEGs) in transgenic 5XFAD mice, ageing mice, and transgenic mice after administration of exogenous Hsp70. (**B**) Restoration of spatial memory in bulbectomized (OBX) mice as indicated by performance in the Morris maze when exogenous Hsp70 is administered. *—*p* < 0.05; ***—*p* < 0.001.

Exogenous Hsp70, as well as endogenous Hsp70, has a strong neuroprotective effect, as demonstrated in several AD models. The addition of exogenous Hsp70 induces microglial activation and promotes Aβ phagocytosis in vitro [187]. Transgenic mice and *Drosophila* flies secreting Hsp70 into intercellular medium have been constructed. In strains obtained by crossing extracellular Hsp70 producers with those carrying *APP* transgenes expressing Aβ and prone to the development of neurodegenerative processes, a pronounced neuroprotective effect has been observed compared to the control cross or wild-type strain [188]. Several investigations [103,142,143,189] have shown that recombinant Hsp70 labeled with Alexa Fluor or radioactive iodine penetrates the brain when administered intranasally and accumulates mainly in the cortex and hippocampus, the structures most severely affected in AD. Sub-chronic intranasal administration of recombinant human Hsp70 has resulted in a decrease in Aβ concentration, activation of neurogenesis, and restoration of cognitive function (particularly spatial memory) in two mouse models of AD, i.e., bulbectomized mice and 5XFAD transgenic mice [142,189]. Furthermore, analysis of the hippocampal transcriptome of 5XFAD transgenic mice intranasally treated with recombinant Hsp70 shows a significant decrease in the expression of genes critical for the development of neuroinflammation, which plays an important role in the development of AD [158,189]. In addition, the expression of genes critical for antigen presentation, in particular, belonging to MHC classes I and II, has been shown to be significantly increased [143]. Transcriptome studies have also shown an increase in the main neurorepair markers and increased activity of neurotransmitter synthesis systems after administration of exogenous Hsp70 (Figure 5). In addition, intranasal administration of Hsp70 has resulted in the activation of genes involved in the regulation of the MAPK cascade [143], which plays an important role in cell proliferation, synaptic plasticity, and memory consolidation [94]. These data are in good agreement with the work presented in Reference [85], which shows the modulating effect of exogenous Hsp70 on the activity of the MAPK cascade. Finally, administration of recombinant Hsp70 leads to reduced Aβ levels in the brain and restored neuron density in bulbectomized and transgenic mice [142,189]. Chronic intranasal administration of Hsp70 to ageing mice results in a slight increase in longevity, as well as an increase in neuron density and synaptophysin levels in the cortex and hippocampus, which indicates the restoration of the synaptic network that degrades with age [190]. Bulbectomized transgenic 5XFAD and ageing mice treated with recombinant Hsp70 have been shown to have greater memory recovery, as revealed in the Morris water maze test (Figure 5) [142,189].

There are several cases when human recombinant Hsp70 or Hsp70 inductors were successfully used in clinics [191,192]. For example, the treatment with Hsp70 was applied to patients with lysosomal storage disease (LSD) which often leads to severe damage of CNS [191]; however, application of Hsp70 in clinics is beyond the scope of this review.

Generally speaking, Hsp70 may play a dual role in memory formation and various types of neurodegeneration described above. First of all, Hsp70 may participate in different memory-related processes exploring its well-known chaperone properties. On the other hand, inducible Hsp70 sometimes found outside the cells at trace concentration, as well as exogenous recombinant Hsp70, may exercise its “chaperokin” properties and serve as a “danger signal” activating innate immunity and other protective cellular systems.

## 8. Conclusions

Hsp70 family members are involved in the formation and maintenance of memory in different ways. First, the stimuli that trigger the stress response and the induction of Hsp70 synthesis also activate the processes of memory and the formation of new synapses. The transcription factor HSF1 promotes not only Hsps production but also the rapid synthesis of synaptic proteins. Furthermore, Hsp70 functions as a molecular chaperone, facilitating the transport and folding of synaptic proteins and modulating the signaling cascades involved in the formation of synapses. Furthermore, Hsp70 (both endogenous and exogenous) protects neurons and synaptic structures from damage under stress (heat shock, hypoxia, etc.). Finally, Hsp70 interferes with the oligomerization and aggregation of proteins prone to amyloid formation, preventing neurodegeneration. Taken together, the accumulated data allow us to consider Hsp70 synthesis inducers and recombinant Hsp70 per se as promising neuroprotectors.

## Abbreviations

AD	Alzheimer’s disease:
APP	amyloid precursor protein;
CaMKII	Ca^2+^/calmodulin-dependent protein kinase II;
cAMP	cyclic adenosine monophosphate;
CRE	cAMP response elements;
CNS	central nervous system;
CREB	CRE-binding protein;
ERK	extracellular signal-regulated kinases;
HS	heat shock;
HSE	heat shock element;
HSF	heat shock factor;
Hsps	heat shock proteins;
JNK	c-Jun N-terminal kinases;
LTM	long-term memory;
LTP	long-term potentiation;
MAPK	mitogen-activated protein kinase;
PKA	cAMP-dependent protein kinase;
PKC	protein kinase C;
STM	short-term memory;
STP	short-term potentiation
